# Chronic treatment with escitalopram and venlafaxine affects the neuropeptide S pathway differently in adult Wistar rats exposed to maternal separation

**DOI:** 10.3934/Neuroscience.2022022

**Published:** 2022-09-13

**Authors:** Miłosz Gołyszny, Michał Zieliński, Monika Paul-Samojedny, Artur Pałasz, Ewa Obuchowicz

**Affiliations:** 1 Department of Pharmacology, Faculty of Medical Sciences in Katowice, Medical University of Silesia, Medyków 18, 40-752 Katowice, Poland; 2 Department of Medical Genetics, Faculty of Pharmaceutical Sciences in Sosnowiec, Medical University of Silesia, Jedności 8, 41-200 Sosnowiec, Poland; 3 Department of Histology, Faculty of Medical Sciences in Katowice, Medical University of Silesia, Medyków 18, 40-752 Katowice, Poland

**Keywords:** neuropeptide S, neuropeptide S receptor, antidepressants, maternal separation

## Abstract

Neuropeptide S (NPS), which is a peptide that is involved in the regulation of the stress response, seems to be relevant to the mechanism of action of antidepressants that have anxiolytic properties. However, to date, there have been no reports regarding the effect of long-term treatment with escitalopram or venlafaxine on the NPS system under stress conditions.

This study aimed to investigate the effects of the above-mentioned antidepressants on the NPS system in adult male Wistar rats that were exposed to neonatal maternal separation (MS).

Animals were exposed to MS for 360 min. on postnatal days (PNDs) 2–15. MS causes long-lasting behavioral, endocrine and neurochemical consequences that mimic anxiety- and depression-related features. MS and non-stressed rats were given escitalopram or venlafaxine (10mg/kg) IP from PND 69 to 89. The NPS system was analyzed in the brainstem, hypothalamus, amygdala and anterior olfactory nucleus using quantitative RT-PCR and immunohistochemical methods.

The NPS system was vulnerable to MS in the brainstem and amygdala. In the brainstem, escitalopram down-regulated NPS and NPS mRNA in the MS rats and induced a tendency to reduce the number of NPS-positive cells in the peri-locus coeruleus. In the MS rats, venlafaxine insignificantly decreased the NPSR mRNA levels in the amygdala and a number of NPSR cells in the basolateral amygdala, and increased the NPS mRNA levels in the hypothalamus.

Our data show that the studied antidepressants affect the NPS system differently and preliminarily suggest that the NPS system might partially mediate the pharmacological effects that are induced by these drugs.

## 1. Introduction

Neuropeptides, which are neuromodulators, are involved in the regulation of a wide range of CNS functions. There is convincing evidence that support the hypothesis that the hypothalamic neuropeptides play an important role in emotionality and the stress response [1]. For many years, intense investigations of neurobiological interplay have been conducted in particular brain areas that have provided data that indicates that non-hypothalamic peptides probably also regulate mood and anxiety and, as a result, might partially mediate the pharmacological effects of antidepressive and anxiolytic drugs. This activity has been attributed to the peptides that are secreted by the brainstem nuclei: neuropeptide Y (NPY), substance P (SP), galanin (GAL), relaxin-3 (RLN-3), phoenixin (PNX), nesfatin-1 (NEFA), neuropeptide Q/spexin (NPQ/SPX) and neuropeptide S (NPS) etc. [2]–[7].

**Figure 1.**
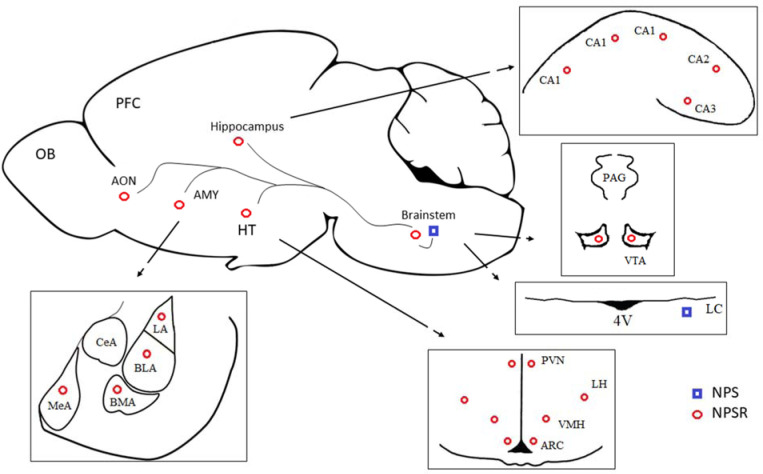
Neuropeptide S (NPS) pathways in the rat brain. NPS is expressed in the brainstem and reaches the limbic system and anterior olfactory nucleus (AON). In the brainstem, NPS is synthesized in the peri-locus coeruleus (peri-LC) (blue squares). The link between the peri-LC and hypothalamic nuclei, e.g., the paraventricular nucleus (PVN), is a pathway that is involved in the expanded regulation of the limbic system and neuroendocrine response to stress, according to Nauta's conceptualization. The neuropeptide S receptors (NPSR) are localized in the ventral tegmental area, hypothalamic nuclei, amygdaloid complex, hippocampus and AON (red circles), according to previous studies [11],[12]. Abbreviations: AMY—amygdala; AON—anterior olfactory nucleus; ARC—arcuate nucleus; BLA—basolateral amygdala; BMA—basomedial amygdala;CeA—central amygdala; CA1-CA3—areas of hypothalamus; HT—hypothalamus; LA—lateral amygdala; LC—locus coeruleus; LH—lateral hypothalamus;MeA—medial amygdala; NPS—neuropeptide S; NPSR—neuropeptide S receptor; OB—olfactory bulb; PAG—periaqueductal grey area; PFC—prefrontal cortex; PVN—paraventricular nucleus; VMH—ventromedial hypothalamus; VTA—ventral tegmental area; 4V—4th ventricle.

Neuropeptide S (NPS) is a 20-aminoacid peptide that is involved in stress responsivity in humans [8] and lab animals [9]. Some data has shown that NPS and the neuropeptide S receptor (NPSR) play a significant role in regulating food intake, anxiety, arousal and fear in experimental models [10]; however, the mechanisms of action of the NPS/NPSR system has not yet been fully recognized. In the rat brain, the expression of the NPS precursor gene is limited tothe brainstem nuclei, whereas projections of the NPS-immunopositive fibers have been detected in various brain regions, including the limbic system [11],[12]. In the classical understanding, this system is recognized as the emotional Papez circle and in more the modern formulation, as Nauta's limbic-midbrain system orthe olfactory-limbic tract (illustrated in Fig. 1) [13]–[15]. The effects of NPS are mediated *via*the NPSR [16], which is found in many areas of the brain with the highest densities in the cortex, thalamus, hypothalamus, subiculum and amygdala [11],[12],[17]. To date, the relationship between the NPS system and neurotransmitters such as dopamine [18],[19], serotonin and norepinephrine [20] has only been revealed in a few studies. The influence of psychotropic drugs on the NPS system is almost unknown. It has been found that antipsychotic drugs up-regulate the NPS mRNA expression in the rat hypothalamus [6],[21].

The majority of studies concerning the role of neuropeptides in the mechanism of action of antidepressants have been focused on the hypothalamic peptides and changes in the activity of the neuropeptidergic signaling in the diencephalon (interbrain), whereas little is known about the significance of alterations in the peptidergic neuronal pathways that project from the brainstem for antidepressive and anxiolytic effects of drugs. Some preclinical studies have suggested that the pharmacological effects of the selective serotonin reuptake inhibitors (SSRIs) are partially mediated by neuropeptides [22] but the effects of the serotonin and norepinephrine reuptake inhibitors (SNRIs) have been rarely investigated in this regard.

The present study is focused on the influence of two antidepressants: escitalopram, a representative of SSRI and venlafaxine, a “flagship” selective inhibitor of the serotonin and norepinephrine reuptake (SNRI) on the NPS system in the rat brain. Escitalopram being citalopram with the R enantiomer removed, is the most selective SSRI. This drug is frequently used in the treatment of mood and anxiety disorders [23],[24]. Escitalopram interacts with both the orthosteric and allosteric sites of the serotonin transporter (SERT) and enhances serotonin transmission, which is thought to be the basis for its therapeutic effects [25]. Recently, evidence that escitalopram alters the NPQ/SPX mRNA expression [26], proopiomelanocortin (POMC) [27] and OX-A [28] was found in the rat hypothalamus. Venlafaxine is frequently used in the therapy of moderate-to-severe depression and generalized anxiety disorder and phobias [29]. This drug, which is an inhibitor of the SERT and norepinephrine transporter (NET), increases the activity of the serotonergic and noradrenergic systems [30]. The relevance of neuropeptidergic signaling to the mechanism of action of venlafaxine is still poorly investigated. Only a few studies have indicated that venlafaxine does not alter the corticotropin-releasing factor (CRF) [31], GAL [32] orOX-A [28] in the rat brain. On the other hand, when given chronically, this antidepressant reversed the increased arginine-vasopressin (AVP) plasma levels in olfactory bulbectomized mice [33].

The early-life period is significant for the proper development of all animals and human beings. Close contact between newborns and their mothers/dams is necessary because the first days of life shape the emotional, physiological and behavioral reactions for life. Adverse events in early life can induce anxiety- and/or depressive-like behaviors [34],[35] and are a major risk for developing psychopathologies in adulthood. Maternal separation (MS) is an experimental model that is frequently used to study the long-lasting consequences of disturbances in maternal care [36]–[38]. However, the mechanisms that are involved in the effect of MS on the neuropsychophysiological development of neonates have not yet been fully recognized. However, it has been shown that MS affects the activity of the neurotransmitter systems (dopaminergic, serotonergic and noradrenergic) [39]–[41] and neurohormones (CRF, adrenocorticotropin-releasing hormone (ACTH) andcorticosterone (CORT) [42]. Some studies have indicated that MS also affects the neuropeptidergic systems: NPY [43], OX-A [28],[44], POMC [45] and NPS [46]. It has been observed that when escitalopram is given chronically, it mitigates some of the behavioral and neurobiological changes that were induced by stressful events in early life [47], whereas the neurochemical effects of venlafaxine in rats that have been subjected to early-life stress have rarely been investigated. A lack of the effect of venlafaxine on the orexin A system in the rat brain has also been reported [28].

Therefore, the aim of the presentpreliminary study was to determine whether given chronically at the same dose escitalopram or venlafaxine alter the NPS system activity in the brainstem-limbic-olfactory areas of the brain of rats that had been subjected to repeated maternal separation (MS) and in non-stressed rats.

## 2. Materials and methods

### 2.1. Animals and ethics approval of research

Female pregnant Wistar rats (2.5 to 3 months old, weighing 250–300 g) that had been purchased from a licensed breeder (Center for Experimental Medicine of the Medical University of Silesia) (offspring of rats from the Charles River, Germany) were used in this study. The rats were adapted to their new conditions at the animal facility of the Department of Pharmacology for one week before the study began. The dams were maintained individually in plastic cages in a room under controlled conditions (12/12 hours light on at 7:00 a.m., ambient temperature of 22 + 2°C and humidity 55 + 10%; food and water were available *ad libitum*). The day of delivery was designated as postnatal day (PND) – 0. The litter size was adjusted to 8–10 pups on PND 1 (an equal number of males and females, whenever possible) and then the litters were randomly assigned to the groups that were exposed to MS or to the control (non-stressed) groups. The dams and the rats after weaning had continuous free access to food because the NPS system regulates the feeding behavior [48]. The study protocol was approved by the Local Ethical Committee for the Care and Use of Laboratory Animals in Katowice (agreement no. 144/2016). All of the procedures were conducted in accordance with the guidelines of the European Directive (2010/63/EU).

### 2.2. Experimental design

In the adult male rats (3 months old) the effects of MS and/or the antidepressant drugs that had been chronically administered on the NPS system were evaluated in the brain structures that are involved in regulating emotional states and stress response. The expressions of the NPS precursor mRNA and NPSR mRNA were analyzed using the qRT-PCR method. The NPS and NPSR immunoreactive neurons were estimated using an immunohistochemical analysis. To avoid the litter influence on the outcomes of the study, the studied groups (control and MS exposed) consisted of rats that had originated from the different litters. Escitalopram (10 mg/kg/), venlafaxine (10 mg/kg) or a 0.9% NaCl solution were injected intraperitoneally (IP) once daily from PNDs 69–90. The study was conducted on six groups of rats (n = 12–14). Escitalopram hydrochloride and venlafaxine hydrochloride (Alembic Pharmaceuticals Ltd, a gift from Adamed, Poland) were dissolved in a 0.9% NaCl solution. The above mentioned non-toxic doses of drugs were established on the basis of pharmacological standards developed in preclinical studies and had been used in experiments previously published in the literature that examined the effect of these medications on the neurotransmittion/neuromodulatory pathways [26],[49]–[51].

Twenty-four hours after the last drug dose was administered (9:00 to 11:00 a.m.), the rats were sacrificed by decapitation and the material for determining the gene expression and immunohistochemical analysis was collected. In order to avoid pre-decapitation stress, the rats were handled shortly prior to decapitation and when a rat was sacrificed, the others were kept outside of the surgery room.

### 2.3. Maternal separation (MS)

The pups were separated from their dams for 360 minutes (9:00 a.m. to 3:00 p.m.) from 2 to 15 PND as was previously described by Mesquita et al. [52]. PNDs 2–15 have been reported as the critical period for the development of a neuroendocrine response to stress [38],[53],[54]. Each litter (in a new plastic cage with fresh bedding material) was taken to an adjacent room where it was placed on a heating mat (temp. 34 °C) in order to maintain a constant body temperature of the pups [55]. The researchers wore medical aprons and latex gloves. The pups from the control litters were not handled and were kept with their dams in the home cages. The rats were weaned on PND 21. Only males were used in this study. In rats, the responses to stress and long-term consequences of MS are gender-dependent. This issue was not the aim of the present study. Nevertheless, the majority of experiments have been focused only on males because outcomes obtained in the evaluation of long-term consequences of MS in adult female rats may be disturbed by estrous cycle phases [56]. Moreover, Dimatelis and colleagues [57] suggested that female rats were resistant to developing depressive-like behavior induced by maternal separation. In our study, male rats were housed in standard plastic cages (52 cm × 31 cm × 19 cm) in groups of four or five rats under controlled conditions as described above.

### 2.4. Total RNA isolation, reverse transcription and quantitative RT-PCR – analyses of the NPS and NPSR mRNA expression

After decapitation (n = 8–10 rats per group), the brains were removed and the brainstem, hypothalamus and amygdaloid complex were dissected according to the stereotaxic atlas of Paxinos and Watson [58]. The isolated brain structures were homogenized using an ultrasound homogenizer (Microson Ultrasonic Disruptor, Misonix Bioz Inc., Farmingdale, NY, USA) in 1 ml of ice-cold *TRIzol*™ Reagent (Life Technologies, Carlsbad, CA, USA). Then, the homogenates were incubated for 10 minutes at room temperature in order to permit the complete dissociation of the nucleoprotein complexes. All of the ribonucleic acids were extracted from the cells according to the typical Chomczynski method [59]. Finally, the supernatant was removed and the RNA pellet was washed twice with 1ml of 75% ethanol and air dried. The RNA extracts were qualitatively evaluated in 1% agarose gel using electrophoresis and quantitatively usingspectrophotometry (Biophotometer, Eppendorf, Hamburg, Germany).The primers and probes for the amplification of the NPS (Forward: 5′-AAAACTCAACCTCATCTTAGC-3′, Reverse: 5′-AAATGAGAAAGTAATCAGGCTTC-3′), NPSR (Forward: 5′-TAATCCTTGCTTTCATCTGC-3′, Reverse: 5′-AGTAGATGAGGGGGTTAATG-3′) and GAPDH (Forward: 5′-GTGAACGGATTTGGCCGTATCG-3′, Reverse: 5′-ATCACGCCACAGCTTTCCAGAGG)mRNA were obtained from Sigma Aldrich (Sigma Aldrich, St. Louis, MO, USA). The total RNA was reverse-transcribed into single-strand cDNA and cDNA copies, which were amplified using a TaqMan One-Step RT-PCR Master Mix Reagents Kit (ThermoFisher, Waltham, Massachussets, USA). The reaction mix (25 µl) contained 1.25 µl of sequence-specific primers and probes, 12.5 µl of RT-PCR Master Mix, 0.625 µl of MultiScribe and RNA-se Inhibitor, 2.5 µl (10 ng) total RNA and 8.125 µl of RNA-se free water. qRT-PCR was performed using an ABI PRISM^®^7700 Sequence Detection System (ThermoFisher, Waltham, Massachussets, USA) (RT: 48 °C-30 min; PCR: 95 °C-10 min, 40 cycles: 95 °C-15s, 60 °C-1 min). The expression of the *NPS* and *NPSR*genes was compared with the expression of the housekeeping gene glyceraldehyde phosphate dehydrogenase (GAPDH). A common method that is used to analyze the relative gene expression data is the protocol that was presented by Livak-Schmittgen [60], which compares the two values in the exponent that represent the normalized expression values for each sample. The results are shown as the relative expression ± SEM. The statistical analysis was calculated based on the raw ΔCt values.

### 2.5. Immunohistochemical analysis of the NPS and NPSR-immunoreactive neurons

Subdural injection of a fixative was performed and after decapitation (n = 4 rats per group), the rat brains were removed and post-fixed in 4% paraformaldehyde for 12 hours and then paraffin-embedded samples were prepared. The brain slices were dehydrated and embedded in paraffin and then sectioned in the coronal plane at a 7µm thickness on a microtome (Leica Microsystems, Wetzlar, Germany) according to the atlas by Paxinos and Watson [58] as follows: hypothalamus and amygdala (−2.00 to −2.80 mm from the bregma); brainstem (−9.60 to −10.08 from the bregma) and anterior olfactory nucleus (4.20 to 6.20 from the bregma). After rehydration, antigen retrieval (in a low pH antigen unmasking solution, Vector Laboratories, Burlingame, CA, USA) and blockage with Hydrogen Peroxide Block (10 min) and Protein Block Reagents (60 min), the brain sections were incubated overnight at 4°C with a rabbit antibody against rat NPS (1:4000, Merck Millipore; Millipore Sigma, *Burlington, MA, USA*) or NPSR (1:3000, Merck Millipore; Millipore Sigma, *Burlington, MA, USA*). Sections (on the same slide) that had been incubated with rabbit IgG instead of the primary antibody were used as the negative controls. Incubation with the primary antibodies or rabbit IgG was followed by the administration of biotinylated anti-rabbit secondary antibodies for 20 min (Abcam, Cambridge, United Kingdom). Finally, 3.3′-diaminobenzidine (DAB) was used (Abcam, Cambridge, United Kingdom) to visualize the cells that expressed specific neuropeptides. Next, some of the slices were incubated in hematoxylin (Vector Laboratories, Burlingame, MA, USA) for 1 minute. The NPS immunoreactive neurons in the brainstem (peri-locus coeruleus) were counted and the NPSR immunopositive cells were visually evaluated in the brainstem (peri-locus coeruleus and raphe magnus nucleus) and were counted in the hypothalamus (the paraventricular, arcuate, ventromedial nuclei and lateral perifornical area), amygdala (the basolateral nuclei) and anterior olfactory nucleus using the ImageJ 1.43 Fiji plugin (National Institutes of Health, Bethesda, USA). The negative control of the analysis revealed that the NPS and NPSR antibodies that were used were specific and selective – the omission of the primary antiserum resulted in a complete lack of immunostaining.

The results are shown as a percentage of the control ± SEM.

### 2.6. Statistical analysis

Statistical analysis was performed using the GraphPad Prism 8.4.3 (GraphPad Software, San Diego, CA, USA). The normality of the data distribution was checked using the Shapiro-Wilk test (significance level α = 0.05). The homogeneity of variance (Levene's test with η^2^ effect type, α = 0.05) was conducted using www.statskingdom.com online software (Statistic Kingdom, Melbourne, Australia). All effects were determined using a one-way ANOVA followed by a *post hoc* Tukey or Kruskal-Wallis test with *post hoc* Dunn's test. A p < 0.05 value was considered to be a statistically significant difference.

## 3. Results

### 3.1. Escitalopram and venlafaxine affect the NPS and NPSR mRNA expression differently in the brainstem, hypothalamus and amygdala of adult rats that have been subjected to MS and non-stressed rats

#### 3.1.1. NPS and NPSR mRNA

In the brainstem, we found significant alterations in the NPS mRNA between the studied groups (H = 11.90, p < 0.05). Nevertheless, MS did not induce any alterations in this parameter.

**Figure 2.**
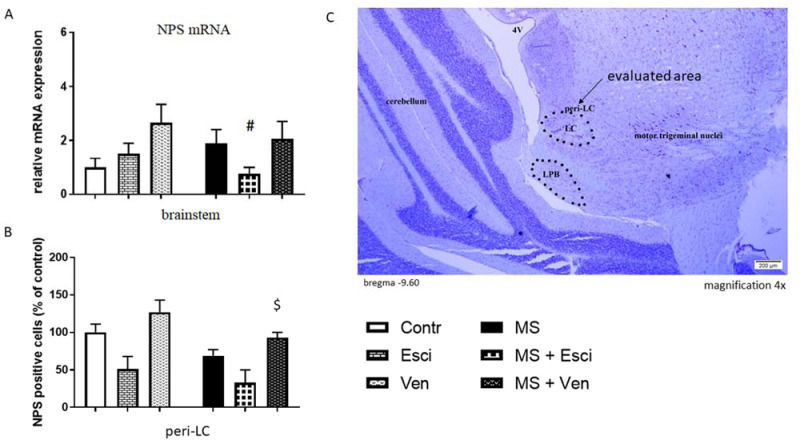
The effects of maternal separation (MS) and/or the long-term administration of escitalopram (10 mg/kg IP) or venlafaxine (10 mg/kg IP) on the relative NPS mRNA expression in the brainstem and NPS-immunopositive cells in peri-LC of adult male Wistar rats. (A) Values are the relative expression ± SEM (n = 8–10 per group). # p < 0.05 vs. MS (*post hoc* Dunn's test). (B) NPS-immunopositive cells (% of control ±) (n = 4 per group); (C) A representative photomicrograph showing the evaluated area – peri-LC in the cresyl violet stain (magnification 4 x). $ p < 0.05 vs. MS + Esci (*post hoc* Dunn's test). Abbreviations: LPB—lateral parabrachial nucleus; peri-LC—peri-locus coeruleus; Contr—non-stressed rats receiving saline (IP); Esci—non-stressed rats receiving escitalopram (IP); Ven—non-stressed rats receiving venlafaxine (IP); MS—rats subjected to MS on PNDs 2–15 and receiving saline (IP); MS + Esci—rats subjected to MS on PND 2–15 and receiving escitalopram (IP); MS + Ven—rats subjected to MS on PNDs 2–15 and receiving venlafaxine (IP).

A decreased NPS mRNA level was detected in the stressed rats that had been treated with escitalopram (p < 0.05, *post hoc* Dunn) while venlafaxine had no effect. The differences between these two drugs were close to statistical significance (p = 0.0555, *post hoc* Dunn) (Fig. 2 A).

The expression of NPSR mRNA was observed in all analyzed structures-brainstem, hypothalamus and amygdala.

In the brainstem, there were differences between the study groups (H = 12.64, p < 0.05).

**Figure 3.**
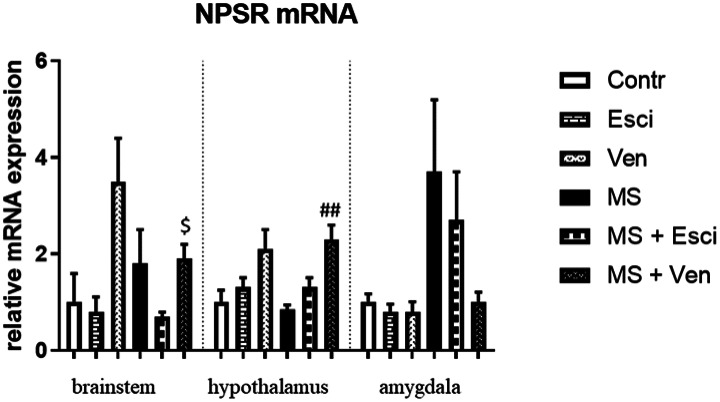
The effects of maternal separation (MS) and/or the long-term administration of escitalopram (10 mg/kg IP) or venlafaxine (10 mg/kg IP) on the relative NPSR mRNA expression in the brainstem, hypothalamus and amygdala of adult male Wistar rats. Values are the relative expression ± SEM (n = 8–10 per group). $ p < 0.05 vs. MS + Esci (*post hoc* Dunn's test); ## p < 0.01 vs. MS (*post hoc* Dunn's test). Abbreviations are explained in the legend for Fig. 2.

MS did not induce significant alterations in the NPSR mRNA expression. Escitalopram induced only a statistically insignificant decrease in the NPSR mRNA, while venlafaxine did not cause any effects. The differences between the NPSR mRNA level in the MS rats that had been treated with escitalopram or venlafaxine were significant (p < 0.05, *post hoc* Dunn). The NPSR mRNA expression was not different in the non-stressed groups (Fig. 3).

In the hypothalamus, we found significant alterations in the NPSR mRNA between the studied groups (H = 18.53, p < 0.01). MS did not affect the expression of the NPSR mRNA. Venlafaxine markedly increased the NPSR mRNA expression (p < 0.01, *post hoc* Dunn). This drug also induced an increase in the NPSR mRNA expression in the non-stressed group, however, this effect was not statistically significant (Fig. 3).

In the amygdala, the differences between the analyzed groups were not significant, because of the high SEM values. MS caused only an insignificant increase in the NPSR mRNA level. Venlafaxine reduced NPSR mRNA expression insignificantly in the stressed rats. No alterations were observed in the non-stressed groups (Fig. 3).

#### 3.1.2. NPS and NPSR-immunoreactive neurons

**Figure 4.**
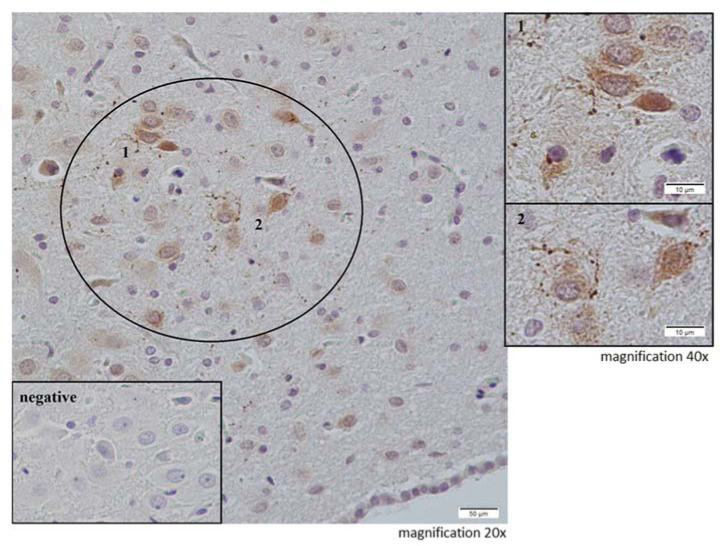
Neuropeptide S in the peri-LC – the representative photomicrographs.

The NPS-immunoexpression was evaluated in the coronal plane (−9.60 to 10.08 mm from the bregma) in some of the brainstem nuclei (Fig. 2 C). A few NPS-positive neurons were found in the peri-LC (peri-locus coeruleus). Other brainstem nuclei that express NPS, e.g., the motor trigeminal nucleus, were not analyzed since they are not involved in emotionality. The NPS-positive neurons are depicted in Fig. 4.

In general, the differences between all analyzed groups were significant in the peri-LC (H = 17.69, p < 0.01).MS caused an insignificant decrease in the number of NPS-immunopositive neurons (69 ± 8% vs. Contr) (Fig. 2 B). This observation was confirmed by the measurement of optical density (OD) (data not shown). The antidepressants caused a tendency toward opposite effects. When compared with the MS group, there was an insignificantly reduced number of the studied cells in the rats that had been treated with escitalopram (48 ± 17% vs. MS) and in turn, venlafaxine induced an insignificant increase (135 ± 7% vs. MS) (Fig. 2 B). In the MS rats, an insignificant increase of OD was observed after long-term treatment with venlafaxine (data not shown). The differences between the effects of venlafaxine and escitalopram were significant in the MS rats (p < 0.05, *post hoc* Dunn).

There was also a decrease in the number of the NPS-immunopositive neurons after treatment with escitalopram (51± 17% vs. Contr) and an increase was induced by venlafaxine (127 ± 16% vs. Contr) in the non-stressed rats (Fig. 2 B). Nonetheless, these results have not achieved the level of statistical significance.The OD measured in the peri-LC in the non-stressed rats that had been treated with escitalopram was decreased insignificantly (data not shown).

The NPSR-immunoexpression was analyzed in some of the hypothalamic areas, the basolateral amygdala and the anterior olfactory nucleus.

**Figure 5.**
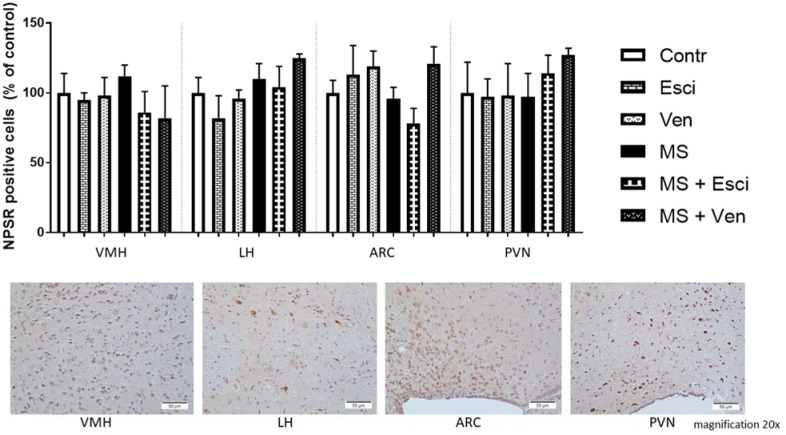
The effects of maternal separation (MS) and/or long-term administration of escitalopram (10 mg/kg IP) or venlafaxine (10 mg/kg IP) on the number of immunopositive NPSR cells in the ventromedial hypothalamus (VMH), lateral hypothalamus (LH), arcuate nucleus (ARC) and paraventricular nucleus (PVN). Values are the percentage of control ± SEM (n = 4 per group). Representative photomicrographs of NPSR immunostaining in the studied subregions in control group. Abbreviations are explained in the legends for Fig. 1 and Fig. 2.

A relatively high level of expression of NPSR was observed in various hypothalamic areas (−2,00 to −2,80 mm from the bregma) such as the ventromedial hypothalamic nucleus (VMH), dorsomedial hypothalamic nucleus (DMH), lateral hypothalamus (LH), arcuate nucleus (ARC), paraventricular nucleus (PVN) and paraxiphoid nucleus (PaXi). In our study, the effects of MS and/or antidepressants were analyzed in four hypothalamic areas: VMH, LH, ARC and PVN. In all of the studied areas, the relative number of NPSR positive cells was not changed in the MS rats. Whereas, it was statistically insignificantly altered by both of the studied drugs in this group of rats. In the VMH, an insignificant decrease was observed in the MS rats after treatment with escitalopram (76 ± 15% vs. MS) or venlafaxine (73 ± 23% vs. MS). In the LH, venlafaxine caused a slight increase in the MS group (114 ± 3% vs. MS) whereas escitalopram reduced the number of this cell population in the non-stressed rats (82 ± 16% vs. Contr). These effects were not statistically significant. In the ARC, there was an insignificant increase (126 ± 12% vs. MS) in the NPSR positive cells in the MS rats that had been treated with venlafaxine. Escitalopram induced an opposite effect (81 ± 11% vs. MS). The differences between escitalopram and venlafaxine were altered insignificantly. In the PVN of the MS rats, both drugs slightly increased the number of NPSR positive cells – venlafaxine (131 ± 5% vs. MS) and escitalopram (118 ± 13% vs. MS). Because of the high SEM values, these results were not statistically significant (Fig. 5). There were no significant differences in the OD (data not shown).

**Figure 6.**
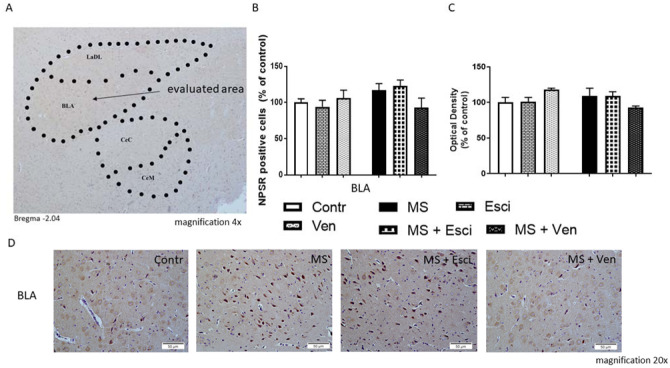
The effects of maternal separation (MS) and/or the long-term administration of escitalopram (10mg/kg IP) or venlafaxine (10mg/kg IP) on the number of immunopositive NPSR cells and optical density in the basolateral amygdala (BLA). (A) A representative photomicrograph of the evaluated area in the amygdaloid complex (magnification 4x) (coronal plane −2.04 from the bregma); (B-C) The NPS-immunopositive cells and optical density (% of control); (D) Representative photomicrographs showing the NPSR immunopositive cells in the BLA (magnification 20×). Abbreviations: CeC—central amygdaloid nucleus, capsular, CeM—central amygdaloid nucleus, medial division, LaDL—lateral amygdaloid nucleus, dorsolateral. The otherabbreviations are explained in the legends of Fig. 1 and Fig. 2.

In the amygdala, alterations in the number of NPSR positive cells were analyzed in the basolateral amygdala (BLA) in which relatively high levels of the NPSR mRNA have been reported. NPSR positive cells were detected in the basomedial amygdala (BMA) but not in the central areas of this amygdaloid complex (the evaluated area is depicted in Fig. 6 A). In the BLA, there were no statistically significant alterations in the studied groups. The MS rats demonstrated a slight increase in the number of NPSR-immunopositive cells (117 ± 9% vs. Contr), however, it was not confirmed by enhancing the OD. In this group of rats, venlafaxine reduced the studied cell population insignificantly (80 ± 13% vs. MS), whereas escitalopram did not induce a similar effect. No alterations were observed in the non-stressed rats that had been treated with the antidepressants (Fig. 6 B-C).

**Figure 7.**
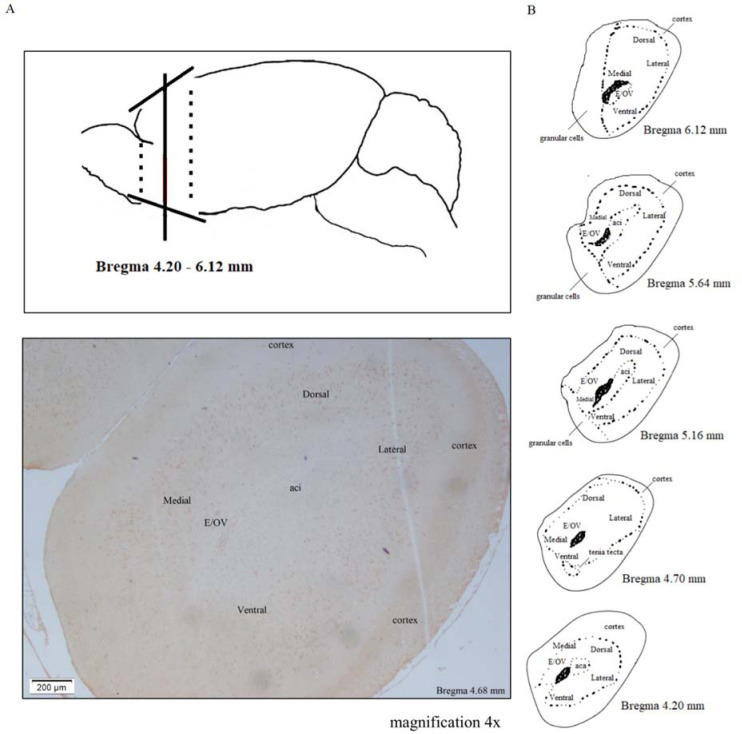
The NPSR-immunopositive cells in the rat anterior olfactory nucleus (AON). (A) Part of the brain including the anterior olfactory nucleus from which the slices were prepared according to the atlas by Paxinos and Watson (2006) (the coronal planes 4.20 to 6.12 from the bregma); (B) A representative photomicrograph showing NPSR expression in control rats (3,3′-Diaminobenzidine–immunostaining, magnification 4×). Abbreviations: aci—anterior commissure, intrabulbar; E/OV—ependyma/olfactory ventricle

**Figure 8.**
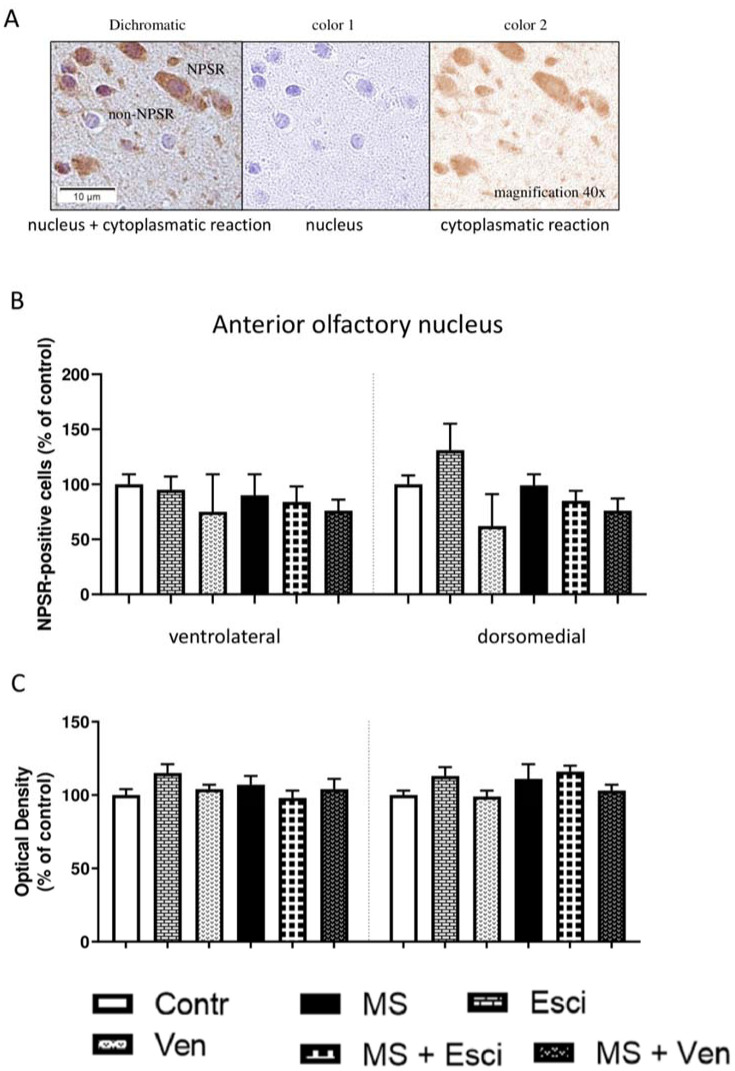
The effects of maternal separation (MS) and/or long-term administration of escitalopram (10 mg/kg IP) or venlafaxine (10 mg/kg IP) on the number of immunopositive NPSR cells and optical density in the anterior olfactory nucleus (AON). (A) A representative microphotograph showing the result of NPSR-cytoplasmatic positive reaction. NPSR protein was not detected in cellular nucleus. Color deconvolution; (B) NPSR-immunopositive neurons in the ventrolateral and dorsomedial areas of the AON (% of control); (C) Optical density (% of control).

In the anterior olfactory nucleus (AON), the NPSR-positive cells were distributed in the ventrolateral and dorsomedial areas (Fig. 7). The cytoplasmatic reaction is illustrated in Fig. 8 A. MS had no effect on the number of these cells in the AON and the antidepressants that had been given chronically did not cause any significant alterations. In rats that had been exposed to MS, venlafaxine did not significantly alter this cell population in the ventrolateral area (86 ± 10% vs. MS) and dorsomedial area (77 ± 11% vs. MS). In the non-stressed rats, escitalopram increased the number of NPSR-positive cells in the dorsomedial area (131 ± 24% vs. Contr), but venlafaxine induced an opposite effect (62 ± 29% vs. Contr). Nonetheless, the differences between the effects of these drugs were not statistically significant, as well as no relevant alterations were noticed in the OD (Fig. 8 B-C).

Abbreviations are explained in the legend for Fig. 2.

## 4. Discussion

In the presented study, we found that in the adult male rats that had been exposed to MS, the studied parameters of the NPS system were not significantly altered; however, there wereinsignificant alterations in NPS and NPSR mRNA expression as well as in the number of NPSR-positive cells in the studied regions of brainstem and amygdala. Previously, we have noticed in the same groups of rats that MS_2-15_360 protocol is well-validated and induces long-term behavioral and neuroendocrine consequences [28]. MS is frequently used as an animal model of early-life stress that induces abovementioned changes in adults that mimic anxiety- and depression-related features in other studies [34],[35]. In our previous paper, we showed that MS caused anxiety-like behavior (estimated in the open-field (OF) elevated plus-maze (EPM) tests) and a tendency towards the development of despair behavior (the Porsolt test) in adults. These behavioral alterations observed in MS rats were associated with overactivity of the HPA axis [28].

When given chronically, escitalopram and venlafaxine affected the NPS and NPSR mRNA differently. Escitalopram down-regulated the NPS and NPSR mRNA expression in the brainstem of the MS rats whereas venlafaxine had no effect. Venlafaxine up-regulated the NPSR mRNA levels in the hypothalamus and induced an insignificant increase in the number of NPSR-positive cells in the studied hypothalamic areas while this parameter decreased insignificantly in the amygdala. The results were diverse depending on the basal or stress condition.

As was mentioned in the Introduction, besides the alterations in some of the neurotransmitter systems [39]–[41],[45],[61] in the adult MS rats, there were also changes in the neuropeptidergic signaling [28],[44],[46],[62],[63].

To date, only one study of alterations in the NPS system in adult male Wistar rats that had been exposed to MS has been published by Bulbul and Sinen [46]. They found some alterations in the NPS release in the BLA and the number of NPS-positive cells in the LC in the brainstem, BLA and hypothalamic PVN. Our presented data that show statistically insignificant alterations in the NPS system in the MS rats are only partially in line with the results of their experiments.

NPS modulates a variety of physiological functions (anxiety, mood, food intake, aversive memory, arousal and wakefulness) [64]. Its ability to diminish the response to fear, anxiety and stress [65] has also been reported in some investigations [66]–[68]. In the presented study, in order to evaluate the effects of escitalopram and venlafaxine on the NPS system activity in MS and non-stressed rats, we focused on a few areas of the brainstem-limbic-olfactory system. Earlier, WalleNauta included the brainstem to limbic system and described a link between the brainstem nuclei and the hypothalamus and amygdala [14],[69].

In the brainstem, MS did not cause any statistically significant alterations in the NPS mRNA and NPSR mRNA expression. Nonetheless, a tendency toward a decrease in the number of NPS and NPSR-positive cells in the peri-LC that was confirmed by the measurement of OD have been found. These data concerning the NPS system in whole brainstem or peri-LC area that were obtained in two different analyses (qRT-PCR and IHC-P) are equivocal. It is known that qRT-PCR estimation provided gene analysis in various areas expressing NPS molecules, including structures that were not connected with emotional states (such as motor trigeminal nuclei). Nevertheless, the alteration detected in the peri-LC suggests the down-regulation of the NPS system in adult rats that have been exposed to MS. Because NPS induces anxiolytic effects, this alteration might be of significance for the anxiety-like behavior that is observed in adult rats exposed to MS [38],[70]. In fact, in the brainstem, the LC, which is the major source of norepinephrine for the forebrain, has been implicated in induction of the stress response [71],[72]. The NPS neurons that are located in the peri-LC are surrounded by noradrenergic neurons. In the brainstem, neuropeptides such as CRF are elements of a complex interaction that has significance for the response to stressors [73],[74] and an interplay between the CRF and NPS has been described [66],[75]. Previously, we suggested overactivation of the HPA axis based on the estimation of peripheral ACTH and CORT levels and following other similar studies. Earlier, Feng et al. [44] detected the increased levels of CRF in the hypothalamus of male MS rats.

In rodents, like noradrenergic transmission, NPS/NPSR signaling is involved in the arousal and exploratory activity [16],[76]. Currently, little is known about the activity of the noradrenergic projections from the LC to the limbic structures in MS rats or about its role in the long-lasting outcomes of MS with regard to mental disturbances. For example, a decrease in norepinephrine turnover in the prefrontal cortex of rats that had been subjected to MS [41], a lack of alterations in the noradrenergic receptors [77] or no changes in the norepinephrine levels in the hippocampus of male rats that had been subjected to MS [78] have been reported.

Using immunohistochemical analysis, we found that although there is no NPSR in the central nuclei of the amygdaloid complex, this protein is present in the basolateral part (BLA) of the amygdala (Fig. 4A), which plays an important role in the regulation of emotional responses including anxiety-related behaviors [79]–[81]. Recently, Grund and Neumann [82] observed that the amygdaloid nuclei are an important brain target of NPS for inducing anxiolytic effects in male rats and that an NPSR-evoked phospholipase C signaling cascade underlies this local anxiolytic effect. Our results showed an insignificant increase in the NPSR expression in the amygdaloid complex and in the number of NPSR-positive neurons in the BLA, which was confirmed by enhancing OD in the BLA of rats subjected to MS. One might suppose that these alterations are compensation for the anxiety-like long-lasting effects that is induced in adult rats by MS [38].

The presented data suggest that NPS in the hypothalamus is not involved in the long-term consequences of MS. The hypothalamus is the main brain structure that is implicated in the regulation of stress and the reproductive and metabolic hormonal axes [83]–[85]. Some studies have indicated that the hypothalamic functions are disrupted in animals that have been subjected to MS [86]–[89]. The hypothalamic NPS system plays an essential role in the regulation of feeding behavior [48]. Because the food intake of rats that were subjected to MS was unaltered (data not shown) in the presented experiments, it seems to indirectly support our suggestion that the hypothalamic NPS is not susceptible to any long-lasting MS effects.

In the presented study, the NPSR mature protein was detected in the anterior olfactory nucleus (AON) for the first time using immunohistochemical staining as is shown in Fig. 7. We did not find any differences in the number of NPSR-positive cells in the ventrolateral and dorsomedial divisions of the AON between the MS and non-stressed rats. The role of the AON – the centrally situated ring-like cortical structure in the tract to olfactory bulb is as yet poorly investigated. The olfactory tract is considered to be a part of the limbic system and connected with mental illnesses [90].

The most important findings of our study are that escitalopram and venlafaxine when administrated chronically have different effects on the NPS system in some of the regions of the brain that have been implicated in the regulation of the emotional states of rats that had been exposed to neonatal MS and non-stressed rats.

For the first time, we found that escitalopram decreases the relative expression of the NPS mRNA and NPSR mRNA in the brainstem of adult MS rats and causes a tendency toward a decrease in the number of NPSR neurons in the peri-LC in the MS and non-stressed rats. NPSRs, which are the target proteins of NPS, are distributed in various serotonin-dependent areas [11],[12],[20], which is why an effect on their modulation might have significance for the mechanism of action of escitalopram. Experimental studies have indicated that when escitalopram is given chronically, it alters the mRNA levels of various neuropeptides in the brainstem [26],[27],[91] and in the other brain structures [28].

In the brainstem, NPS is co-localized with the excitatory neurotransmitters and neuromodulators, including glutamate, acetylcholine, GAL, enkephalin and CRF [72]. A few mechanisms could mediate the suppressive effect of escitalopram on the NPS system in the brainstem. In the MS and non-stressed rats, escitalopram when administrated chronically, it reduced the activity ofthe HPA axis, which is regulated by CRF [28],[51]. When SSRIs are given chronically, they cause a desensitization of the serotonergic 5-HT1A autoreceptors in the brainstem[92] and like fluoxetine causes compensatory changes in the serotonergic 5-HT1A heteroreceptors in the diencephalon (interbrain), which are involved in the activation of the HPA axis [93],[94].Because CRF activates the noradrenergic and NPS neurons in the LC [75],[95], the down-regulation of the HPA axis activity that is induced by escitalopram mightalso mediate its suppressive effect on the NPS system in the brainstem. On the other hand, the peri-LC,in whichNPS neurons are abundantly expressed, receives a dense innervation of serotonergic neuronal projections except for the noradrenergic innervations [96],[97].Escitalopram after long-term administration, increases the availability of serotonin in the synapses [29] and as a result causes adaptive changes in the 5-HT1 and 5-HT2 receptors despite a lack of binding to the 5-HT receptors [29],[98]. Adaptive alterations in the serotonergic transmission, especially in the dorsal raphe [99], are followed by changes in the reaction of LCto noxious stimuli [100] because the activated serotonergic system reduces the activity of the noradrenergic neurons in the LC *via* many targets [101]. It has been shown that when escitalopram is used in the same dose as in our study, it decreases the firing-rate of theLC neurons [102].It might be supposed that the NPS neurons in the LC, which are noradrenergic neurons, are inhibited by the serotonergic system [100],[103], and that therefore, escitalopram may inhibit NPS neurons in the peri-LC *via* an increased activity of the serotonergic transmission in the dorsal raphe. However, it cannot be excluded that other systems such as the dopaminergic [6],[104], glutamatergic [101] and GABAergic systems [105] are indirectly involved in the effect of escitalopram on the NPS neurons. Further studies are required to better recognize the mechanisms of NPS system regulation, which is important because it is known that a modulation in the NPS system activity has an indirect impact on the monoaminergic transmission in various areas of the brain [20].

On the basis of the present knowledge about the role of NPS, the tendency toward a decrease in the activity of the NPS system in the peri-LC does not support the anti-anxiety effect of escitalopram that has been observed in MS rats [28] but may have significance for other its effects. NPS and noradrenergic transmission play equivalent roles in the regulation of some processes. NPS and norepinephrine promote arousal and wakefulness [66]–[106] and regulate the stress reaction [76],[107] and emotionality [10],[108].

In contrast to escitalopram, when venlafaxine was given chronically, it did not induce any alterations in the NPS system in the rat brainstem. This drug increases the activity of the serotonergic and noradrenergic systems in a dose-dependent manner [30]. Nevertheless, some preclinical studies indicate that even in the low to moderate doses, this drug may induces adaptive changes in the noradrenergic system [50],[109].

Next, venlafaxine effects on the 5-HT1A receptors in the dorsal raphe neurons are unequivocal [110],[111]. Moreover, we previously reported that venlafaxine did not alter the HPA axis activity in non-stressed rats and in adult rats that have been exposed to MS [28], and also others indicated that this medication did not affect the synthesis of CRF [31]. These differences in neurochemical effects induced by venlafaxine and escitalopram might be a reason for their different impact on the NPS system in the brainstem, which is regulated by the activity of the serotonergic and noradrenergic systems and CRF. The impact of venlafaxine on the neuropeptide systems in the rat brainstem is as yet poorly investigated. No alterations were found in the GAL transcripts after chronic treatment with this drug [32].

Our study revealed that venlafaxine but not escitalopram altered the expression of the NPSR mRNA in the hypothalamus of the non-stressed and MS rats. An increase in the NPSR mRNA that was induced by venlafaxine was significant in the MS rats. This effect was convergent with results ofthe immunohistochemical analysis, which indicated a tendency towards an increased number of NPSR-positive neurons in three hypothalamic areas: the LH, ARC and PVN. The lack of the effects of escitalopram on the NPSR mRNA levels and the number of NPSR-positive neurons in the hypothalamic areas might be caused by cross-talk between peptidergic systems such as NPQ/SPX [26], POMC [27] and OX-A [28], which are up-regulated after chronic treatment with this antidepressant [112],[113].

Escitalopram had no effect on the NPSR mRNA levels in the amygdala. However, in the rats that had been subjected to MS, venlafaxine insignificantly decreased the NPSR mRNA levels in the amygdala and induced a similar tendency in the number of NPSR-positive neurons in the BLA of the amygdaloid complex. The BLA noradrenergic system is important for the function and activity of this structure [114],[115]. An intra-BLA infusion of NPS increases the activity of the noradrenergic system in the BLA [116]. One might suppose that in the amygdala, venlafaxine could have the effects on the NPS/NPSR system at the result of its impact on the noradrenergic system. The effect of venlafaxine on NPS that has been reported might have significance for its anti-anxiety activity that was previously observed in the elevated plus maze test in MS rats [28].

Previous studies revealed the effects of antidepressant medications on the olfactory tract [117],[118]. It was demonstrated that a one-week treatment with fluoxetine improved neurogenesis in the olfactory bulbs of male mice [117] and that the administration of subchronic citalopram decreased the density of the serotonergic fibers in the rats [118]. Hence, we hypothesized that escitalopram or venlafaxine may induce changes in the NPSR activity in this area of the brain of MS rats.The long-term administration of both of the antidepressants that were studied did not cause any significant changes in the AON – the nucleus that is localized in the tract leading to the olfactory bulb.

## 5. Conclusions, limitations and potential further investigations

Taken together, our preliminary results provide evidence that when escitalopram and venlafaxine are given chronically, they affect the NPS system differently in the brain of adult male rats that had been exposed to neonatal MS. For the first time, it has been shown that escitalopram decreased the NPS mRNA and NPSR mRNA level in the brainstem accompanied by a tendency toward a decrease in the number of immunopositive-NPSR neurons in peri-LC in the MS rats whereas venlafaxine up-regulated the NPSR mRNA in the hypothalamus and to some extent increased the number of immunopositive-NPSR neurons in the studied hypothalamic areas. There is a prior study indicating that escitalopram and venlafaxine given in the same doses affected NPS system differently as statistical analyze confirmed. Recent clinical studies suggested that venlafaxine should be used in the psychiatric practice in the high doses, because low doses did not give satisfactory clinical effect [119],[120]. It is a very important aspect of psychiatric pharmacology. Different significant effects of the studied drugs did not support theoretical consideration that venlafaxine in low dose should be treated as SSRI.

Our novel preliminary findings suggest that the NPS system in the brainstem and amygdala is vulnerable to MS and may be involved in its long-lasting consequences (such as anxiety-like behavior and altered response of the HPA axis), which were presented in our previous paper [28].

It should be pointed out that there are some limitations to our study. Generally, the results of the immunohistochemical analysis of the number of NPSR and NPS-immunopositive neurons were not statistically significant. According to Bonapersona et al. [121],[122] and Button et al. [123], the lack of sufficient power to detect experimental effect is an emerging issue in preclinical studies that should be considered as a limitation and the cause of preliminary character of result. Today, increasing sample sizes to increase statistical power is problematic for both ethical and practical reasons. These preliminary novel findings should be treated as an initial step in the pharmacological studies focused on the influence of escitalopram and venlafaxine on theneuropeptidergic signaling in MS rats. It is known that the mRNA levels do not reflect the protein levels and that when they are evaluated in the whole structure, they do not reflect changes in the subregions (in our study: the brainstem vs. the peri-LC). In order to better recognize the putative role of NPS in the mechanism of action of escitalopram and venlafaxine, the activity of this system in other brain regions (e.g., hippocampus, prefrontal cortex, basomedial amygdala) should also be evaluated in lab animals in different models of stress and depression. Moreover, further research is required in order to study the relationship between NPS and the monoaminergic systems, which is poorly investigated despite the wide distribution of the NPS-immunopositive fibers in the limbic system. The lack of data made it difficult to perform an analysis of the probable mechanism of the alterations in NPS system that were induced by the chronic treatment with escitalopram or venlafaxine. Our pilot findings suggest that the pharmacological effects of the studied antidepressants might be partially mediated by the NPS system. Further investigations are needed to evaluate the role of the NPS system, which is an interesting pathway that is connected to the brainstem, mesolimbic structures and olfactory tract, in the mechanism of action of antidepressants.
