# Supplementary material for: Chronic treatment with escitalopram and venlafaxine affects the neuropeptide S pathway differently in adult Wistar rats exposed to maternal separation
Source: AIMS Neurosci. 2022 Sep 13;9(3):395–422. doi: 10.3934/Neuroscience.2022022 (PMC9581731; doi:10.3934/Neuroscience.2022022)
Supplement: Supplementary file 1 [file neurosci-09-03-022-s001.pdf]

AIMS Neuroscience,9 (3): 395–422.
DOI:10.3934/Neuroscience.2022022
Received:13 June 2022
Revised:20 August 2022
Accepted:05 September 2022
Published: 13 September 2022

http://www.aimspress.com/journal/neuroscience

Research article

Chronic treatment with escitalopram and venlafaxine affects the neuropeptide S pathway differently in adult Wistar rats exposed to maternal separation

Miłosz Gołyszny[1,*], Michał Zieliński[1], Monika Paul-Samojedny[2], Artur Pałasz[3] and Ewa Obuchowicz[1]

[1] Department of Pharmacology, Faculty of Medical Sciences in Katowice, Medical University of Silesia, Medyków 18, 40-752 Katowice, Poland
[2] Department of Medical Genetics, Faculty of Pharmaceutical Sciences in Sosnowiec, Medical University of Silesia, Jedności 8, 41-200 Sosnowiec, Poland
[3] Department of Histology, Faculty of Medical Sciences in Katowice, Medical University of Silesia, Medyków 18, 40-752 Katowice, Poland

* **Correspondence:** Email:miloszgolyszny@o2.pl; Tel: +48 32 2088522; Fax: +48 32 2523835.

Supplementary materials

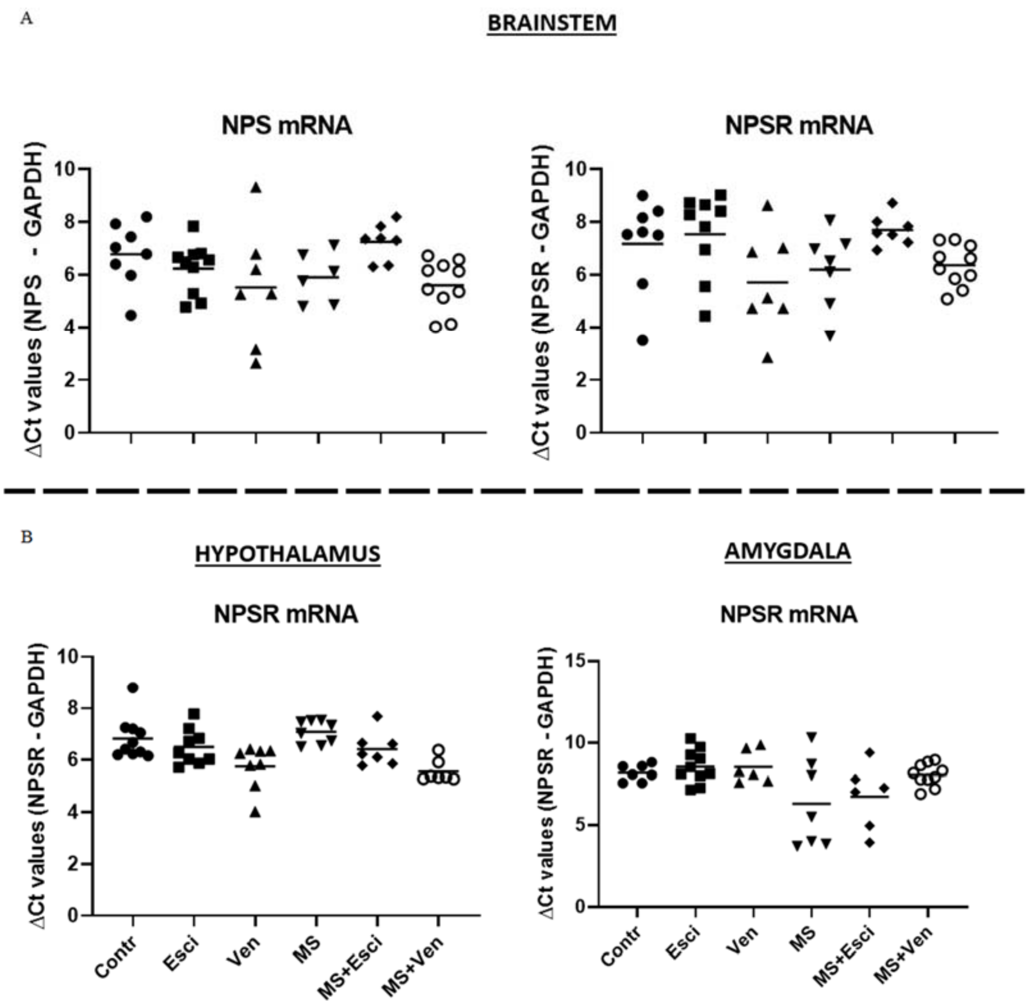

Figure S1. The individual data points from ΔCt values obtained in qPCR.(A) Plots for each groups in the NPS and NPSR mRNA level analyses in brainstem. (B) ΔCt values from each groups in the NPSR mRNA expression analysis in the hypothalamus and amygdala.

AIMS Press

©2022 the Author(s), licensee AIMS Press. This is an open access article distributed under the terms of the Creative Commons Attribution License (http://creativecommons.org/licenses/by/4.0)
